# Nursing Behaviour in Alpacas: Parallels in the Andes and Central Europe, and a Rare Allonursing Occurrence

**DOI:** 10.3390/ani15070916

**Published:** 2025-03-22

**Authors:** Jana Marešová, Tersia Kokošková, Eliška Tichá, Tamara Fedorova

**Affiliations:** Department of Animal Science and Food Processing, Faculty of Tropical AgriSciences, Czech University of Life Sciences Prague, Kamýcká 961/129, 165 00 Prague, Czech Republickokoskova@ftz.czu.cz (T.K.);

**Keywords:** allosuckling, nursing of crias, maternal behaviour, nursing duration, *Vicugna pacos*

## Abstract

Understanding maternal behaviour is essential for good animal husbandry; yet, little scientific information currently exists regarding nursing behaviour of alpacas (*Vicugna pacos*). Moreover, allonursing (the nursing of non-filial offspring) has only been documented in captive non-domesticated guanacos. This study aimed to (1) describe the nursing behaviour of alpacas within traditional husbandry systems in their place of origin, and in foreign conditions, and to (2) investigate the occurrence of allonursing in alpacas. A total of 1899 nursing bouts were observed in the Andes and Central Europe. Additionally, 102 Andean farmers were asked if they observed allonursing in their herds. Nursing behaviour was similar at both locations, with most of the nursing bouts initiated by crias and terminated by dams. However, several significant differences between localities were noted, such as the less frequent sniffing of crias by Andean dams. Crias’ age and sex did not affect the percentage of nursing bouts initiated or terminated by the dams. Rare cases of allonursing were recorded in Central Europe (n = 5), and 18.6% of surveyed Andean farmers reported allonursing in their herds. This study provides insight into alpaca nursing and allonursing behaviour, which may contribute towards their management both in their place of origin and farther.

## Simple Summary

Understanding alpaca maternal and nursing behaviour is important for improving farming practices; yet, little currently research exists. Allonursing describes a situation in which a female nurses non-filial offspring. In South American camelids, this behaviour has only been documented in captive non-domesticated guanacos, and not in domestic alpacas. This study observed the nursing behaviour of alpacas in their native region, the Andes, and in Central Europe, to identify similarities, differences, and the occurrence of allonursing. Nursing behaviour was similar across locations, with most nursing bouts initiated by crias (i.e., alpaca young), and terminated by dams. The dams preferred similar nursing positions. However, Andean dams sniffed their crias less often while nursing. Crias’ age and sex did not affect the percentage of nursing bouts initiated or terminated by the dams. Allonursing was not observed in the Andes but occurred rarely in Central Europe, but 18.6% of Andean farmers reported observing allonursing in their herds. These findings enhance our understanding of alpaca maternal care and may help to improve farm management and animal welfare.

## 1. Introduction

South American camelids (SACs), including alpacas (*Vicugna pacos*), are indigenous to the Andes and are well-adapted to the local climate [1]. They are traditionally managed under pastoral systems [2]. However, approximately half of the population of domestic SACs is located outside of South America [3], and they are popular in other parts of the world, including Europe [4,5,6]. These populations located outside of their place of origin are facing different environmental conditions [1] and management [6,7], which can lead to differences in the behaviour of SACs kept in non-native areas compared with those in the Andes [8,9]. Understanding animal behaviour, including maternal and nursing behaviour, is necessary for herd management, especially with regard to animal welfare, and the successful breeding and rearing of young animals. However, studies which describe the nursing behaviour of alpacas, or SACs in general, are lacking or rather superficial. For example, the suckling behaviour of llamas (*Lama glama*) has only been investigated using six dams by Pouillon et al. [10].

The mother–young relationship plays a crucial role in the early life of newborn ungulate offspring [11,12]. Sniffing and licking of the young is crucial for the formation of the mother–young bond [13,14] and for the identification of filial offspring [13,14,15,16]. Even though camelids do not lick their young, sniffing plays an important role in camels (*Camelus domedarius*) [17] and SACs [16,18]. Nursing provides essential nutrition for the young that depend on milk, especially in areas where no supplementary feeding is available [19]. The young of SACs, or crias, are dependent on milk from their mothers for several months after the birth [20]. Traditional weaning is usually practiced when crias are 4–6 months; however, lactation in SACs can last up to 14 months [21]. On average, alpacas spend 1.99% of their time nursing their crias [18].

In ungulates, nursing is often initiated by the dam in the first weeks after birth [22,23,24,25]. When the young become older, suckling is initiated mostly by them [23,25,26] by approaching the dam [26,27]. In llamas, the majority of suckling bouts appear to be initiated by the crias as well [10].

Dams frequently orient the young into an antiparallel nursing position; i.e., the dam and young are standing in opposite directions [24,28,29,30], which makes individual recognition, e.g., by sniffing, easier [24,28]. The antiparallel nursing position is also preferred by llamas [10]. The parallel and perpendicular positions typically make the identification of the young more difficult in ungulates [31] and are, thus, often seen when allosuckling occurs, i.e., the suckling of milk from a non-filial female [28,31,32]. The occurrence of the perpendicular position seems to be less frequent in the older young [33].

The duration of nursing is likely influenced by the dam [34], when she leaves or drives the young away [26,27,35]. When the young is older and weaning is imminent, nursing is more frequently terminated by the dam than by the young [26,27,36,37]. The nursing duration becomes shorter as the young gets older [23,26,27,37], but the nursing duration can also be prolonged in some cases as the young grows older [24,38]. Less total time spent suckling and decreased suckling frequency in older young were also confirmed in llamas [10]. Nursing bouts initiated by the dam seem to be longer than those initiated by the young [24,36] and nursing bouts terminated by dams tend to be shorter [29,39]. The nursing position can also affect the nursing duration, which tends to be longer in an antiparallel position [28,40,41].

The sex of the young can also affect the nursing duration. Nursing tends to be mostly longer in males [22,23,24,28,42,43] but some studies found that the nursing duration was longer in female young [44,45] or did not observe any influence of sex [36,40]. The nursing duration and frequency in male and female young can also vary and differ depending on the young’s age [45,46] as a potential result of dam–young conflict [45] or different nutritional needs and rates of weight gain in male vs. female young [23,47].

The influence of climate, environment, or management on nursing behaviour is not currently well-described in domestic livestock. Nursing behaviour studies in livestock have focused on restricted nursing (e.g., [48]), and farrowing or an enriched environment in sows (e.g., [49,50]). Only a few studies on non-domesticated species have demonstrated that nursing bouts can be less frequent and longer in more challenging environmental conditions with a higher predation risk [24,40]. On the contrary, farmed deer dams with less resources nursed young more often [51].

Although there is a lack of information about the filial nursing behaviour of camelids, allosuckling has been observed in dromedaries [46], captive Bactrian camels (*Camelus bactrianus*) [28], and non-domesticated guanacos (*Lama guanicoe*) kept on farms for two or three generations after being captured from the wild [16,52]. This behaviour occurs among domestic and wild ungulates [31,47,53]. Allosuckling offers various benefits to the non-filial young, such as an increased rate of weight gain or compensation for nutrient deficiencies in their mother’s milk [16,53,54]. On the other hand, allosuckling also brings with it a large range of imminent dangers, both for the young and for the dam, which includes large energy expenditures for the lactating female, the risk of agonistic behaviour of the dam towards the young, and pathogen transmission [47,55].

Captive conditions [31,47] and a larger litter size [56] can also increase the frequency of allosuckling. Up to 62% of dams of captive guanacos [57] and 67% of Bactrian camel dams [28] perform allonursing, i.e., the nursing of a non-filial offspring. Allosuckling and allonursing behaviours are explained by several hypotheses which have been reviewed by several studies [47,53,58]. The allosuckling duration is also often shorter than the filial sucking duration [28,30,41,43,59] but some studies did not report any differences in the nursing duration between filial and non-filial young [16,60].

Due to the lack of scientific information available, this study aimed to characterise and compare the nursing behaviour of alpacas kept under traditional conditions in the Andes and on farms in the non-native environment of Central Europe, and, furthermore, to investigate the occurrence of allonursing in alpacas, as this behaviour has not been previously described in domestic SACs.

## 2. Materials and Methods

The nursing behaviour of alpacas (*Vicugna pacos*) was observed on farms at two locations (across four counties), in the Andes (Ecuador and Peru), and in Central Europe (Poland and Austria). The observations took place on local farms and did not interfere with normal operations at these farms. The observers did not interact in any way with the observed animals.

### 2.1. Study Areas

#### 2.1.1. The Andes—Ecuador

Four herds in the Chimborazo province were chosen for observations. Each herd consisted of 40 to 60 alpacas. The herds contained 11, 11, 16, and 28 nursing dam–cria pairs. The observations were conducted in two periods: from 28 May to 20 July 2021 (autumn and winter, dry season) and from 15 April to 26 May 2022 (autumn, dry season).

In the observed alpaca herds, dams were kept together with males during the whole year and were usually mated for the first time at the age of 2 years. Weaning of the crias took place at approximately 6.5 months after birth, and, thus, the crias were between 1 to 5 months old at the time of the observations. The herds were placed on the pasture during the day, usually from 8 a.m. to 3 p.m., and the observations of nursing behaviours were performed during this time, when the alpacas were on the pasture. Pasture size was approximately 100 ha. Water was provided by natural resources on the pasture. During the night, the animals were in small enclosures with a shed. They did not receive any supplementary feeding.

Herds were located with an average altitude of 3900 m a. s. l. The annual rainfall of the Ecuadorian páramos is 1000 to 3000 mm or more [61]. The average temperature is 0 to 12 °C with frost and snow occurring. The precipitation during the period of the observations was between 65 to 70 mm [62]. The weather conditions during the observations were variable, from sunny days to very rainy days.

#### 2.1.2. The Andes—Peru

One alpaca herd located in Pampa de Toccra (Arequipa province) was observed in Peru. This area is part of the Salinas y Aguada Blanca National Reserve. The herd had approximately 200 females, of which ca. 100 had crias. The observations were conducted during two periods: from 16 June to 22 June 2021 (winter, dry season) and from 4 June to 1 July 2022 (winter, dry season).

Dams with crias were separated from the males outside of the breeding season, and, thus, also at the time of the observations. The weaning age was approximately 7.5 months. The crias were between 1 to 7 months old during the observation period. Males and females are usually included into the breeding system at the age of 2 years old. Animals were on pasture of approximately 150 ha in size, from 8 a.m. to 6 p.m., and they shared the pasture with wild vicuñas present in this area. No water was available on the pasture during the day. During the night, animals were in an enclosure with a shed, where the water was provided.

The herd was situated at an altitude of 4400 to 4500 m a. s. l. The annual precipitation varies from 350 to 450 mm and is concentrated within the rainy season, which is from December and March [63]. Observations took place during the dry season, and there was no rain during the observation period. The average annual temperature is 3 to 5 degrees Celsius [64].

#### 2.1.3. Central Europe—Poland and Austria

Repeated observations of alpacas in Central Europe were conducted in one Austrian farm (Vöcklabruck district) and three Polish farms (in Pulawy, Ciechanów, and Crakow districts) from 29 June to 5 September 2022 (summer). Total herd sizes varied from 20 to 30 animals per herd, depending on the location, observation period, and herd management. Several dams gave birth during the observation period. In total, 35 dam–cria pairs, divided into 6 herds, were observed.

During the period of the observations, the crias were between 1 and 14 months old. Dams with crias were separated from adult males. Animals were released onto their pastures between 7 and 9 a.m. The herds were rotated between different pastures, each ranging in size from 0.14 to 0.27 ha. All animals had stables where they spent nights, the hottest parts of the day, and, if there was heavy rain. All animals had access to hay and fresh water.

Mean temperatures in Austria and Poland during the observation period were around 18 and 20 °C, respectively, and monthly rainfall varied from 195 to 222 mm and from 60 to 92 mm, respectively [65]. The weather was mostly sunny with several rainy days during the observations. However, there were extremely high temperatures (up to 33 °C) during a few days of observations in Austria.

### 2.2. Data Collection

The nursing data were collected using the all-occurrence sampling method [66] and by two observers (one in the Andes and one in Central Europe). The observers underwent joint training for the observation of nursing behaviour before the start of the research and they shared the same methodology of data collection. To minimise the influence of the observers on the observed alpacas, the animals were observed from a distance of at least 5 m, without major movements of the observers, who were silent during observation.

When dams started nursing, the nursing duration was measured by a stopwatch. Nursing bouts longer than 5 s were considered as successful [32]. Termination of nursing was considered after 10 s of interruption [28]. Initiator and terminator of nursing (dam/cria), sniffing the cria by dam during nursing (yes/no), and the way of initiation and termination were recorded for each nursing bout. Detailed description of the behaviour is presented in the Table 1. The position of the suckling cria towards the nursing female was evaluated as parallel (animals standing in the same direction), antiparallel (animals standing in opposite direction), or perpendicular (cria standing from the side, ca. 90 degrees).

The observations were conducted at time ranges which were suitable for the local farms and their management. They were conducted between 8 a.m. and 3 p.m. in three Ecuadorian herds, and the last herd was observed between 8 a.m. and 1.30 p.m. In Peru, observations were carried out between 8 a.m. and 5 p.m. In Central Europe, observations were carried out from 8 a.m. to 4 p.m. The herds were mostly observed without using binoculars, except for longer distances. Nursing behaviour was observed on pastures in the Andes. In Central Europe, the majority of the observations were conducted on the pastures as well. However, if a herd was enclosed for some part of the observational period due to weather conditions or herd management, the observations continued in stables.

The individual identification of animals and kin relationships could not be determined in most animals in Ecuador and Peru due to the high number of animals present in one herd, the fact that no individual marking was used, and only a very basic register system of the herds is used. Thus, all nursing bouts where just one cria was suckling were considered as filial nursing. Data about nursing bouts were collected from as many animals as possible but recoding of all nursing bouts was impossible because of the high number of dams in Peru. The individual identification of nursing animals in Central Europe was possible, and, thus, the names of the nursing dam and suckling cria were recorded. Sex and age of the suckling crias were also individually recorded in the Central European herds.

Lastly, 44 Ecuadorian and 58 Peruvian farmers were asked if they noticed any occurrence of allonursing in their herds. This question was answered through face-to-face interviews (n = 91) or self-administered questionnaires (n = 11).

### 2.3. Data Analysis

Only nursing bouts longer than 5 s were used for the data analysis. Nursing bouts identified as non-filial were not included in the data analyses because of their rare occurrence (n = 5), so only nursing bouts which were considered as filial were analysed. Given the dataset size and data distribution, parametric statistical methods were applied. The statistical analysis was performed in Statistica, version 13 (TIBCO Software Inc., San Ramon, CA, USA). The level of significance used throughout the analyses was *p* < 0.05.

A dataset of crias aged 1 to 7 months from both the Andes and Central Europe was used for the analysis of nursing behaviour and for comparison of both locations. A dataset of crias of all ages from Central Europe, where the exact age and sex of crias were known, were utilised to test the effects of cria age and sex.

Categorical data, like location (the Andes vs. Central Europe), nursing position, sniffing by dam, initiator and terminator of nursing, and the way of initiation or termination, were analysed using frequency tables and tested using Pearson’s Chi-square test. Differences in nursing duration, according to the categorical variables mentioned above, were tested using Student’s *t*-test and ANOVA. No significant differences were found in the nursing duration of alpacas in Peru and Ecuador (t (923) = 1.64, *p* > 0.05), so these data are presented together as locality “Andes”. No significant differences in nursing duration of alpacas in Poland and Austria were found (t (972) = 1.39, *p* > 0.05), so these data are presented together as locality “Central Europe”.

Generalised linear models (GLMs) were utilised for testing the effects of location, position of cria, terminator, sniffing by dam, and their interactions on nursing duration. For the GLM, the effect of cria age (treated as a continuous variable) and sex were assessed, and nursing dam was used as a random effect. Initiator of nursing was not included in the model because almost all nursing bouts were initiated by the cria (see the Section 3. Results).

Cria age data were also categorised into 4 groups (1–2 months for crias up to 61 days old, 3–5 months for crias 62–152 days old, 6–12 months for crias 153–365 days old, and crias old 366 days or more) and used as a categorial variable to compare the mean nursing duration of cria in different age categories and to test the percentage of nursing bouts initiated or terminated by dams, and the way of initiation and termination.

## 3. Results

### 3.1. Nursing Behaviour of Alpacas and Differences Between Locations

**Figure 1:**
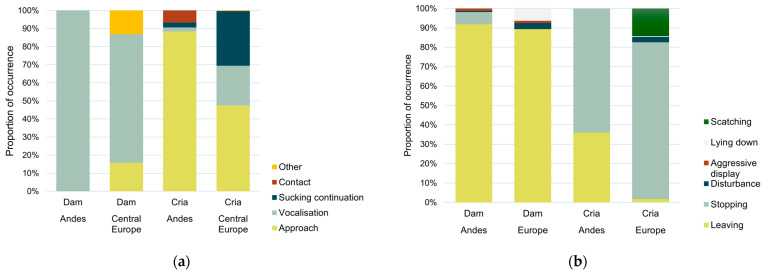
(**a**) The way of nursing initiation according to the nursing initiator and location; and (**b**) the way of nursing termination according to the nursing terminator and location.

Nursing was initiated more frequently by crias (99.14% and 96.10% of nursing bouts in the Andes and Central Europe, respectively) than by dams (χ^2^ (1) = 18.51, *p* < 0.001). The way of initiation was dependent on the initiator (χ^2^ (4) = 303.73, *p* < 0.001). Crias initiated nursing mostly by approach, and dams mostly by vocalisation (Figure 1a).

Nursing was terminated more frequently by dams (59.35% and 51.85% of nursing bouts in the Andes and Central Europe, respectively) than by crias (χ^2^ (1) = 10.82, *p* < 0.05). The way of termination depended on the terminator (χ^2^ (5) = 1222.38, *p* < 0.001). Crias mostly just stopped suckling, whilst mothers terminated nursing by moving away (Figure 1b). The nursing duration results in the Andes and Central Europe are presented in Table 2.

Nursing duration was significantly shorter in Central Europe than in the Andes, when tested without consideration of other factors like sniffing or nursing position (t (1897) = 4.99, *p* < 0.001). However, the GLM revealed that the location (the Andes vs. Central Europe) itself was insignificant and interactions between location and sniffing by the dam, or location and position of the cria played the most significant roles in the nursing duration (GLM: F (14) = 41.92, *p* < 0.001; Table 3).

**Figure 2:**
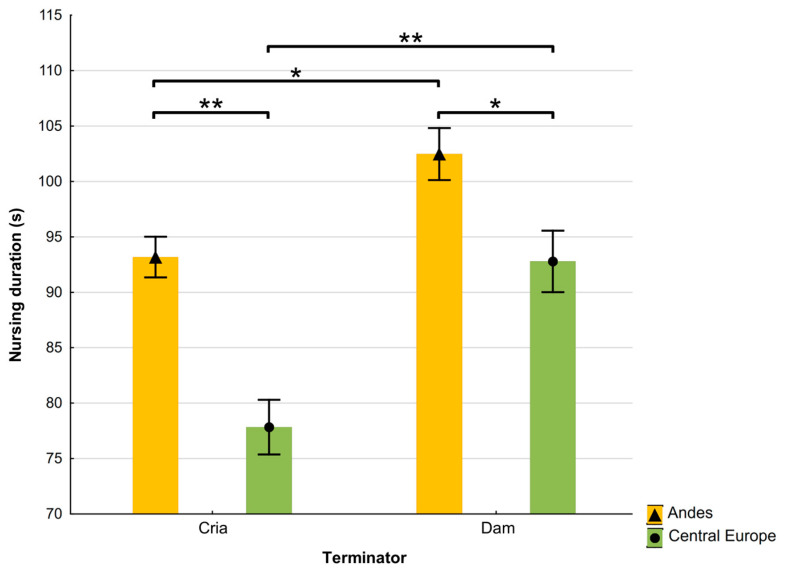
Mean ± SE nursing duration when terminated by alpaca dams and crias, in the Andes and Central Europe (asterisks mark significant differences, * *p* < 0.05, ** *p* < 0.001).

The nursing duration was not affected by the initiator in the Andes; however, it was significantly longer when initiated by the dam than by the cria in Central Europe (112.45 ± 7.79 s vs. 83.92 ± 1.91, respectively; t (972) = 2.97, *p* < 0.05). As shown in Figure 2, the mean nursing duration when terminated by the dam was significantly shorter in both locations.

**Figure 3:**
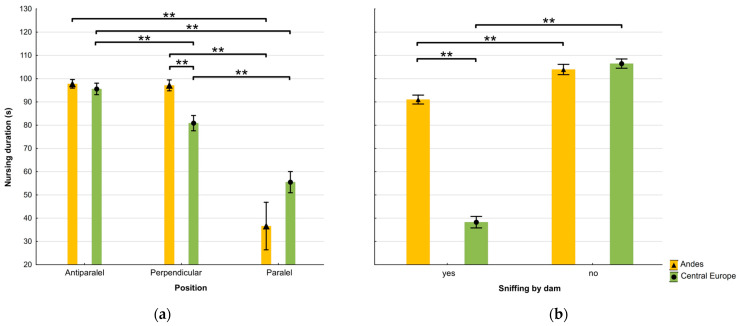
(**a**) Mean ± SE nursing duration of alpacas in the Andes and Central Europe according to different nursing positions; and (**b**) mean ± SE nursing duration in the Andes and Central Europe according to whether the dam sniffed the cria or not. Asterisks mark significant differences, ** *p* < 0.001.

The preferred nursing positions in the Andes and Central Europe were antiparallel (56.76% and 52.26% of nursing bouts, respectively) and perpendicular (42.38% and 33.88%, respectively). The parallel position was significantly less frequent (χ^2^ (2) = 117.18, *p* < 0.05), especially in the Andes (0.86% vs. 13.86% in Central Europe). Likewise, the mean nursing duration was significantly shorter in the parallel position (Figure 3a).

Dams sniffed crias during nursing significantly less often in the Andes than in Central Europe (45.95% vs. 68.58% of nursing bouts, respectively; χ^2^ (1) = 99.52, *p* < 0.001). The mean nursing duration was significantly shorter when the mother did not sniff the cria. However, when the dam sniffed the cria, no significant difference between locations in nursing duration was found (Figure 3b).

### 3.2. Effect of Cria Age and Sex

**Figure 4:**
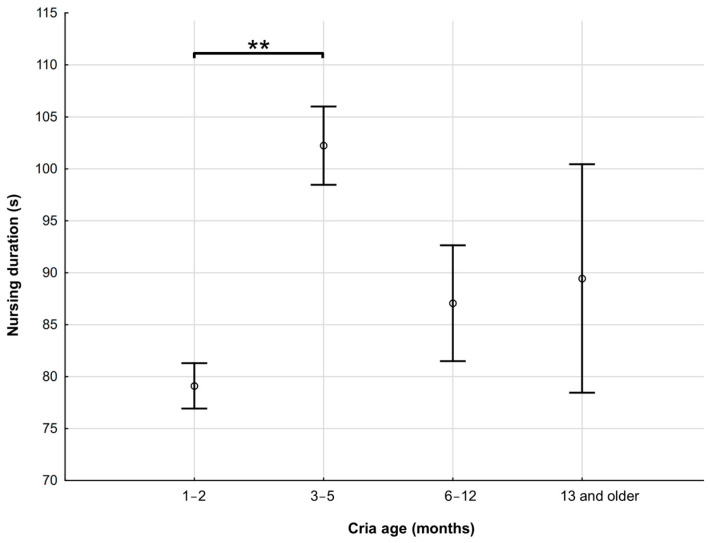
Mean ± SE nursing duration of alpacas across different age categories of crias in Central Europe (asterisks mark significant differences, ** *p* < 0.001).

Cria age and sex did not significantly affect the percentage of nursing bouts initiated or terminated by dams. The way of initiation by crias was also not affected by the age of the cria. However, dams initiated nursing more often when they had crias of up to 2 months of age, and nursing was initiated mostly by vocalisation (57.50% of all case when nursing was initiated by dams; *p* < 0.05). The way of nursing termination of both crias and dams did not significantly change with cria age. Sex did not play a significant role in the way of nursing initiation or termination. The mean nursing duration of crias in different age categories is presented in Figure 4.

**Figure 5:**
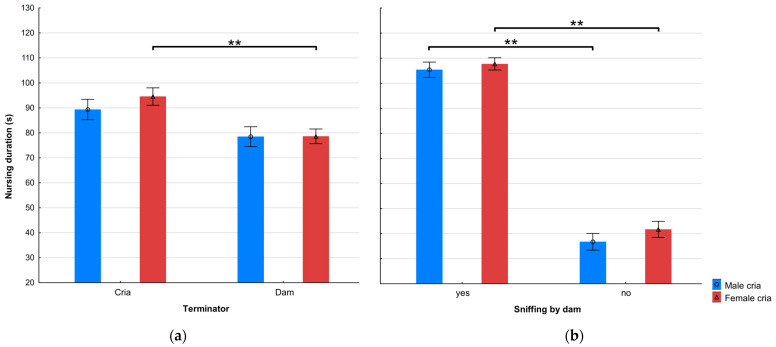
(**a**) Mean ± SE nursing duration of alpacas as affected by the terminator and sex of cria; and (**b**) mean ± SE nursing duration as affected by whether the dam sniffed the cria or not, and cria sex. Asterisks mark significant differences, ** *p* < 0.001.

The model did not show a significant effect of cria age on nursing duration or its interactions with factors like the nursing position, terminator, nor the presence of sniffing by the dam (GLM: F (37) = 16.69, *p* < 0.001). The nursing duration of female crias when terminated by dams was significantly shorter than those terminated by the female crias themselves (t (656) = 3.50, *p* < 0.001). Detailed results are presented in Table 4 and Figure 5.

### 3.3. Occurrence of Allonursing

No allonursing was confirmed by observations in the Andes. The interactions of dams and another (second) cria during nursing were observed only eight times (seven times in Ecuador and only once in Peru) during the observations in the Andes, but none of those started to suckle. The dam typically drove the other cria away and then sniffed the sucking cria. However, 19 out of 102 farmers (18.63%) reported that they saw allonursing in their herds recently. Most of these farmers (n = 18) were from Peru, while only one farmer reported allonursing in Ecuador.

Allonursing was observed in Central Europe during the study. Allonursing was confirmed in 0.38% of successful nursing bouts (i.e., five cases of allonursing performed by four dams), with a mean ± SE duration of 47.2 ± 39.5 s. All cases of allonursing were initiated by the crias, and the filial cria was suckling from the dam at the same time. All non-filial nursing bouts were complemented by sniffing the dam. The perpendicular position was observed in 80% of allonursing cases, which were also terminated by the dam. The majority (80%) of allonursing bouts occurred in the stables. Moreover, nine attempts of crias to allosuckle were observed.

## 4. Discussion

This study characterised the nursing behaviour in alpacas in the Andes and Central Europe, revealing notable similarities at both locations despite differences in climate, pasture sizes, and husbandry practices. In both locations, nursing was initiated predominantly by crias, similar to the reports of other studies [23,25,26]. Dams initiated nursing, accompanied by vocalisation, more often in crias of up to 2 months of age. The frequent vocalisation of the dam for a few days after parturition likely helps to develop the dam–young relationship, as previously described in camels [17] and alpacas [67]. The initiation of nursing by crias, mostly by approaching, is consistent with the results from observations of horses [26,27].

The significantly longer nursing duration when initiated by the dam than by the cria aligns with previous research [22,24,36]. Considering that the most nursing bouts initiated by dams occurred during the first two month of lactation when milk production reaches its peak in SACs [21], the main motivation of dams to initiate and perform longer nursing bouts may be obscured by their aim to empty the udder [24], and their support of the growth and health of their crias [68]. The release of oxytocin, which is connected with the establishment of the dam–young bond and stress reduction [69], also facilitates nursing behaviour in dams. However, a longer nursing duration of bouts initiated by the dam was observed only in Central Europe, not in the Andes, where the nursing duration was not affected by the initiator. This finding can be influenced by the poorer nutrition in the Andes, especially in Peru, where the feeding sources are limited during the dry season [70,71]. Dams may have less milk when lacking nutrients, as reported in camels [72], and less need to get rid of the milk by longer nursing bouts. Contrarily, from the same reasons, alpaca’s crias in the Andes are more dependent on nutrition from their mothers. However, precise conclusions cannot be drawn without an analysis of the nursing frequency and rejection rate, which could provide more comparative results. Unfortunately, these analyses were not possible in this study.

Nursing bouts were terminated mostly by dams, as reported in other studies [26,27,35,36,37]. The most common way of nursing termination was leaving, with rare occurrences of aggressive displays (Figure 1) [26,27,35]. However, most other studies do not describe the ways of nursing initiation and termination, complicating the comparison of results. Nursing bouts terminated by the dam were significantly shorter in both locations, which corresponds with previous findings [29,34,39]. This result supports the assumption that nursing is directed by dams [34]. Dams should decide whether, how often, and how long they nurse their offspring to balance their “costs” of nursing. This conflict increases when the young get older [73] which usually results in a shorter nursing duration [23,26,27,37]. Cria age was not a significant factor affecting nursing duration in the current study but the age of crias was only determined in Central Europe, where resources available for the females were almost unlimited, and so the dams’ need to achieve the nutritional independence of their offspring [73] was not as urgent. This could also explain the prolonged nursing of crias of up to 14 months old.

In the present study, no differences between male and female crias’ nursing behaviour were found, with the exception of the shorter nursing durations of female crias when nursing was terminated by the dam than when it was terminated by the female crias. Female crias may take better advantage of unlimited nursing opportunities, as female young are usually more active than male young, as found in camels [17] or sheep [74]. According to some studies, nursing tends to be longer for male young [28,42,43]; other studies found that the nursing duration was longer for female young [44,45]. These differences are explained by many theories like the faster growth rates of males and higher needs of nutrients [23,37,47], unrelenting suckling in male young [45], or different investments of dams to male and female offspring [45,75]. Male young could also suckle the milk faster [23]. However, the results of the present study did not show any differences between male and female crias in this regard.

Interactions between location and sniffing by a dam, or location and the crias’ position, were identified as more important factors affecting the nursing duration (Table 3). This finding could be associated with the risk of allosuckling. As found in the present study, the duration of nursing was significantly shorter without the dam sniffing the cria [24,42]. The nursing duration did not differ at both locations when dams sniffed their crias. This suggests that smell plays an important role in the identification of crias and maternal behaviour in alpacas [18,35,67]. However, the sniffing behaviour of dams can be quite variable in individual dams and herds as reported in guanacos [57]. The current study supports that alpacas prefer the antiparallel and the perpendicular positions for nursing in both locations. The parallel position makes identifying the young more difficult [30], which could explain why this position was significantly less frequent in the current study. The nursing duration of alpacas was significantly shorter in the parallel position, which is the same trend as in llamas [10] or giraffes [40]. However, in Central Europe, the parallel position was seen more frequently (13.86%) than in the Andes (0.86%). This could result from environmental differences, such as the lack of predators on farms in Central Europe, which allow dams to nurse their crias out of their sight. Supplementary feeding on European farms may make dams more tolerant of the risk of allosuckling [28], increasing their likelihood of accepting the parallel nursing position. However, the allonursing occurrence reported by the Andean farmers is probably not connected with the provision of supplementary feeding in the Andes. The provision of supplementary feeding to alpacas in the Andes is very rare, with only 2.9% of farmers providing it [76]. The pasture size or stocking density could also affect the proportion of nursing positions. The need for young identification does not have to be so strong in smaller herds. Ungulates might recognise herd members just by vision [77] and may, therefore, be able to identify where non-filial offspring occurs in small herds. But this prediction is not aligned with the finding that the nursing duration was the shortest when European dams did not sniff their crias. Sniffing the young during nursing is not always related to individual identification but also to the strengthening of the mother–young bond [13,14]. Therefore, shorter nursing bouts without sniffing the young could indicate that the dams were less receptive to their offspring at that time.

The occurrence of allonursing in alpacas was confirmed within the current study. To the best of our knowledge, this behaviour has not been previously described in domestic SACs. Although allosuckling attempts were observed in both locations, successful allonursing was only observed in Central Europe. This behaviour was far less frequent than reported for farmed guanacos [57], Bactrian camels [28], captive fallow deer [60], or river buffalo [78]. This suggests that allonursing in alpacas is present but could be very rare. Allonursing occurs frequently in species producing litters, when the costs for non-filial nursing are low [56], which is not a case of SACs, as a typical monotocous species [79]. Thus, rare allonursing occurrences in alpacas are not surprising; however, why some monotocous species, including camelids [28,57], allonurse, and others rarely or not at all, remains unclear. Herd sizes and most management practices, including feeding, were comparable between camels [28] and European farms with alpacas. Nevertheless, the incidence of allonursing differed significantly. However, the incidence of allonursing varied considerably across herds, even within the same species and under similar conditions [16,28].

Although allonursing was not observed in the Andes, it cannot be completely ruled out due to the high number of animals in the herds, which made the identification of filial dam–cria pairs more difficult. The nursing of non-filial crias without the simultaneous nursing of filial cria was confirmed by Zapata [57] in guanacos. However, this was not seen in Central Europe in the present study, where non-filial crias always joined the filial nursing. As revealed from the questionnaires, some farmers reported that they had seen allonursing in their herds before. However, this behaviour also may have occurred in the small enclosures where alpacas were kept after returning from the pasture, and, thus, during the hours when observations were not made in the present study. This assumption is also supported by the observations in Central Europe, where the majority of allonursing cases were observed in the stables. Thus, the stocking density may affect allonursing, as dams may have more difficulty recognising their own cria, thus increasing the incidence of allonursing [30]. However, the stocking density in allonursing guanacos [57] was lower than in European farms with alpacas.

All cases of allonursing and allosuckling attempts were initiated by the crias when the filial cria was present near the dam simultaneously in the current study. It is possible that the crias used the presence of the filial young to increase their success of suckling [28], which supports the milk theft hypothesis [32,53,57]. This hypothesis is based on the assumption that the young wants to steal the milk from the non-filial dam, which refuses it after its recognition [31,53], as described in guanacos [57] and camels [28]. It seems that young in the wild tend to steal the milk exclusively when the filial young are present [59,80]. However, young in captivity are also successful without the presence of filial offspring, as confirmed in camels [28], guanacos [57], and other species [32,54], which could mean that dams with unlimited resources could be more tolerant to non-filial offspring [28,41]. But many factors and hypotheses explaining allosuckling behaviour exist [31,47,56], and, considering that the present study confirmed only 5 cases of allonursing, and detailed analyses could not be applied, an in-depth discussion of these factors and hypotheses is unfortunately out of the scope of this study.

Allonursing brings implications to animal husbandry and welfare [47]. Allonursing dams face higher nutritional needs [16], and, thus, the supplementary feeding of allonursing dams may be considered. The dam’s body condition has typically been studied solely as a predictor of allonursing [16,57]. Unfortunately, studies in ungulates do not focus on the body condition of allonursing dams at the end of the lactation period. In general, the body condition of SACs is not greatly affected by lactation [21]; however, camel dams that perform allonursing may quickly decline in body condition [81]. Camel dams allonursed up to three young at one time and allonursing reached up to 35% of all their nursing bouts [28]. Costs connected with the rearing of more than one young, followed by a greater loss of body condition, is clearly demonstrated in ewes [82]. Furthermore, the filial young of allonursing dams could suffer from a lack of milk and slower growth [54,78,83], although other studies did not confirm this finding [84]. Thus, these risks should also be considered in herds of SACs with a high incidence of allonursing, but this was not the case in the current study on alpacas. Contrarily, allosuckling young can improve their nutrition and gains [16,84], and, when weaker or orphaned young are combined with dams which perform allonursing, they can be reared without artificial feeding [81,85].

Further research on the nursing behaviour of domestic SACs, including 24 h observations, and analysing the nursing frequency, rejection rate, etc., is recommended. Furthermore, factors which affect allonursing, such as different husbandry practices, stocking density, and supplementary feeding, should be investigated in alpacas in order to further understand the causes of allonursing [47], especially when the observed rates in alpacas were lower than those reported in other camelids, like guanacos [16,57] or Bactrian camels [28].

## 5. Conclusions

This study provides new insight into the nursing behaviour of alpacas in the Andes and Central Europe. It revealed remarkable similarities in this behaviour in two distinct locations with diverse environment and management conditions. However, differences in nursing duration were found at both locations based on factors such as nursing position or sniffing behaviour. These results contribute to the knowledge of maternal behaviour in domestic SACs and may help farmers monitor and evaluate the nursing behaviour of their animals. The influence of environmental and management factors on SAC nursing behaviour requires further investigation.

The study also confirms the occurrence of allonursing in alpacas, probably as the first scientific report of this behaviour in domestic SACs. Allonursing was observed in the alpacas in Central Europe, although with rare occurrences. Although this study did not observe allonursing in alpacas in the Andes through direct observations, the presence of allonursing was reported by local farmers. However, further detailed research is needed to better understand allonursing and the factors affecting its occurrence in SACs.

## Figures and Tables

**Table 1:** Description of alpacas’ behaviour observed before, during, and after nursing.

Behaviour	Definition
**Way of nursing initiation**	
Approach	Animal approach by walk or run
Contact	Animal performed any physical contact with the second animal before nursing, mostly touching by nose or jumping on dam
Suckling continuation	Situation when cria terminated sucking and started a new bout longer than 10 s after, without any contact or interaction with dam
Vocalisation	Any sound produced before nursing
Other	Mostly standing up or following the second animal that participated in nursing
**Sniffing behaviour during nursing**	
Sniffing by the dam	Dam sniffs the cria’s body
**Way of nursing termination**	
Stopping	Animal terminated the nursing/sucking without firm movement or interaction
Aggressive display	Kicking, spitting, or pushing away
Scratching	Animal started to scratch instead of nursing/sucking
Lying down	Animal lay down on the ground
Disturbance	Animals were disturbed by an external factor

**Table 2:** Mean ± SE, median, minimum, and maximum nursing duration in alpacas in the Andes and Central Europe.

	Number of Nursing Bouts	Mean ± SE Nursing Duration (s)	Median Nursing Duration (s)	SD	Minimum Nursing Duration (s)	Maximum Nursing Duration (s)
Andes	925	96.96 ± 1.46	97	44.29	9	424
Central Europe	974	85.04 ± 1.87	87	58.26	5	300

**Table 3:** Factors affecting nursing duration in alpacas, analysed by GLM.

Effect	df	F	*p*
Location	1	1.02	n. s.
Position of cria	2	7.01	*p* < 0.001
Terminator	1	19.69	*p* < 0.001
Sniffing by dam	1	107.17	*p* < 0.001
Location × Position of cria	2	6.78	*p* < 0.05
Location × Terminator	1	5.00	*p* < 0.05
Position of cria × Terminator	2	0.71	n. s.
Location × Sniffing by dam	1	149.57	*p* < 0.001
Position of cria × Sniffing by dam	2	2.12	n. s.
Terminator × Sniffing by dam	1	2.22	n. s.

n. s. = non-significant, *p* > 0.05.

**Table 4:** Effect of alpaca cria sex and age on nursing duration in Central Europe, analysed by GLM.

Effect	df	F	*p*
Nursing dam (random effect)	23	3.06	*p* < 0.001
Cria age (days)	1	1.26	n. s.
Position × Cria sex	4	0.69	n. s.
Terminator × Cria sex	2	11.29	*p* < 0.001
Sniffing by dam × Cria sex	2	119.53	*p* < 0.001
Position × Cria age (days)	2	1.97	n. s.
Terminator × Cria age (days)	1	0.01	n. s.
Sniffing by dam × Cria age (days)	1	0.23	n. s.

n. s. = non-significant, *p* > 0.05.

## Data Availability

The data are available upon request from the corresponding author.

## Abbreviations

The following abbreviations are used in this manuscript:

GLM	Generalised linear model
m a. s. l.	Meters above sea level
SACs	South American Camelids
